# Effect of a classroom-based intervention on the social skills of pupils with intellectual disability in Southwest Nigeria

**DOI:** 10.1186/s13034-016-0118-3

**Published:** 2016-09-02

**Authors:** Yetunde C. Adeniyi, Olayinka O. Omigbodun

**Affiliations:** 1 Centre for Child and Adolescent Mental Health (CCAMH), University of Ibadan, Ibadan, Nigeria; 2 Department of Child & Adolescent Psychiatry, University College Hospital, Ibadan, Nigeria; 3 Department of Psychiatry, College of Medicine, University of Ibadan, Ibadan, Nigeria

**Keywords:** Social skills, Intellectual disability, Curriculum

## Abstract

**Background:**

Studies have demonstrated that social skill interventions and classroom supports are effective for pupils with intellectual disability. Such interventions have been demonstrated to reduce the risk of developing mental disorders, majority of which have their onset during the period of youth. Most young people with intellectual disability in low-resource settings do not have access to interventions that would enable or enhance their participation in society. The aim of this study was to investigate the effect of a social skills training for pupils with intellectual disability attending a special school in Southwest Nigeria.

**Methods:**

Thirty pupils with mild to moderate intellectual disability participated in the study. Utilising the *Explore* social skills curriculum, teachers were trained to give lessons to the participants 3–4 times a week for 8 weeks in their classrooms. Social skills level of participants was assessed with the Matson evaluation of social skills for individuals with severe retardation (MESSIER) at baseline and immediately after the intervention. Paired t tests, Wilcoxon signed-rank test, Mann–Whitney U test and the Kruskal–Wallis Test were used to assess for pre and post intervention changes in social skills scores and analysis of changes in social skills across socio-demographic variables at p < 0.05.

**Results:**

The mean age of the participants was 15.70 ± 1.89 years. At baseline, 18 of the participants (63.3 %) had moderate social skills impairment, 2 (6.7 %) had none or minimal impairments and 10 (30 %) had severe impairments. At the end of the intervention, there was a 20 % reduction in the number of participants in the severe social skills impairment category and 13.3 % increase in the number of participants in the ‘none or minimal’ social skills category. The mean pre and post- intervention total social skills scores were 126.63 ± 17.91 and 135.97 ± 20.81 respectively with a mean difference of 9.34 (t = 3.71; p = 0.001).

**Conclusion:**

The social skills of pupils with intellectual disability who participated in this study improved significantly during the 8 weeks the *Explore* social skills curriculum was administered. Advocacy should be made for the development and incorporation of social skills curricula into routine teaching of pupils with developmental disabilities.

## Background

Deficits in social skills are critical components of intellectual disability (ID) [1, 2] and researchers have conclusively demonstrated that individuals with intellectual disability have impaired social skills [3, 4]. Social skill deficits are related to many important personal and social outcomes in individuals with ID [5]. For example, many individuals with intellectual disability have less social skills than their same-age peers and are less able to use cognitive social behaviours [6]. Social skills are those behaviours that provide individuals with the skills necessary to interact effectively with others, recognise and respond to social cues, apply appropriate responses in specific situations, avoid interpersonal conflicts, and adjust to simple and complex situations [7]. Greater social skills deficits have been linked to more severe intellectual disability and problems in verbal and nonverbal communication [8]. This can result in isolation of the individuals in social situations [9, 10], lower levels of acceptance from peers and teachers [11–13] and significant social disadvantage and exclusion [14]. Individuals with intellectual disability often have difficulties making and sustaining friendships, and their friendship are characterized by less warmth and closeness and less positive reciprocity than the friendships of normally developing peers [15]. This difference in ability to develop friendships has been attributed to poor social skills development [15].

The impact of poor social skills could be severe during the period of adolescence [16], because at this time, the young person is coping with a rapidly changing mind and body and at the same time coming to the awareness of being different from peers for the first time [17]. Difficulties developing social relationships have been found to impact affective development, resulting in loneliness [18, 19], depression and suicidal ideation [20–22]. These social and mental health problems, in turn, impact the life adjustment of pupils, and result in a higher likelihood of dropping out of school, and even engaging in aggressive and criminal behaviours [17, 23]. In addition, social skills have been demonstrated to be important for a successful transition to adult life for young people with disabilities [24].

Interventions that focused on improving the social skills of young people with intellectual disability tended to improve their participation and ability to cope in the community [9, 23, 25, 26]. Moreover, such interventions have been demonstrated to reduce the risks of developing mental health problems [27–29]. Social skills instructions, and peer support arrangements have been demonstrated to be effective among young people with intellectual disabilities [23, 25, 26, 30]. These instructions have demonstrated the potential to increase independence, probability of successful interaction, and social competence of pupils with intellectual disabilities within school settings [26].

Pupils need social skills to learn effectively in school settings [31] and many ideas for teaching social skills have been developed to support learning and can be found in learning curricula and resources [23, 32]. It has also been shown that helping pupils learn social skills is a proactive approach to minimizing the impact of disabilities on school success [33]. Many methods are being adopted in the training of social skills to pupils with intellectual disability such as role-play, video modelling and photo-based directions [26, 33], curricula and instruction adaptations [23, 25, 34, 35].

Despite the fact that a higher proportion of persons with intellectual disability live in low and middle-income countries [36, 37], very few interventional studies focused on improving outcomes exist in these settings. The few research studies on children and adolescents with intellectual disability in sub-Saharan Africa tend to determine prevalence rates [38].

With the aim of devising culturally appropriate, low intensity, easily to administer interventions to improve outcomes for young persons with intellectual disability, this study investigates the effect of a teacher administered classroom-based curriculum on the social skills of pupils with moderate to severe intellectual disability.

## Methods

### Study location

The study was carried out at the Home School for Handicapped Children, Ibadan, Southwest, Nigeria. It was established in 1964 by the State government to cater for the learning needs of children and adolescents with intellectual disability. The school is an outreach institution of the Centre for Child and Adolescent Mental Health, with which both authors are affiliated. Mental health professionals from this centre pay regular visits to the school to offer on-site consultation, in-service training and support for staff. The school admits children ages 10–19 years from all the six states in the Southwest geopolitical zone of Nigeria, thereby providing services to an estimated population of 27,581,992 [39]. The State’s Ministry of Education coordinates the admission process into the school. As a part of the admission process, pupils have a basic medical assessment carried out by any medical doctor based in a government-owned hospital. An important admission criterion into the school is a diagnosis of intellectual disability which is often made by medical doctors working within government-owned hospitals, this diagnosis is often based on information from caregivers and clinical findings. The children often do not receive appropriate psychological evaluation due to unavailability of both human and material resources to carry out the evaluation. Hence, the children are arbitrarily placed in the three classrooms in the school, designated as ‘educable’, ‘trainable’ and ‘profound’ mentally retarded classes by the school administration. The school runs both a day and boarding service and uses a similar school calendar as the mainstream schools in the state; each school calendar year consists of three terms and one term lasts for 12 weeks. The school closes at the end of each term, and the pupils return to their different homes. The day pupil (there was only one pupil in the day service at the time of study) comes to school from home and returns back home daily. Although each classroom has a maximum capacity for 20 pupils, the number of pupils in the classroom is mostly dictated by the available hostel accommodation.

There is a 40-bed capacity hostel located within the same compound as the school. The hostel was running in full capacity as at the time of study. There were six carers looking after the pupils in the hostel on a shift arrangement. Two carers run a day or night shift at every point in time and assist the pupils with activities of daily living such as self-care, getting ready for school and feeding. The carers had a maximum of 12 years of education but with no special training for the care of children with disabilities. Each of them had spent an average of 5 years as carers in the boarding section of the facility.

### Current teaching facilities

Teachers in the school reported that they utilise the same teaching packages as is used by mainstream schools in the district. They adapt this for their use in the school. They also indicated that they did not have teaching aids such as charts, pictures, and graphs. Majority of these teachers had no training in special education. Aside from the three classrooms used for academic sessions and a hostel facility, there is a very large open field the size of half a football pitch where the young people run around and play. There are no other facilities.

### Participants

There were a total of 40 pupils in the hostel during the period of the study. Only 29 out of the 40 pupils were attending classes and they were included in the study. The remaining 11 of the residents remained within the residential facility and could not attend classes because they had “profoundly mentally retarded” with other associated medical disorders such as severe physical disability and poorly controlled epilepsy, hence were excluded from the study. The only pupil in the day service participated in the study bringing the total number of participants to 30. The 30 participants were between the ages of 12–19 years (mean = 15.70, SD 1.89) and 53 % were males. Ninety-seven percent of participants (29) lived in the residential facility located within the school premises. Other characteristics of the participants are shown in Table 1.

**Table 1:** Socio-demographic characteristics of the participants (N = 30)

Variable	Frequency (%)
Age (years)	
12–14	10 (33.3)
15–19	20 (66.7)
Gender	
Male	16 (53.3)
Female	14 (46.7)
Religion	
Christianity	23 (76.7)
Islam	7 (23.3)
Family type	
Monogamy	20 (66.7)
Polygamy	10 (33.3)
Marital status	
Married	19 (63.3)
Not married	11 (36.7)
Father’s level of education	
No formal education	10 (33.3)
Primary	11 (36.7)
Post Primary	9 (30.0)
Mother’s level of education	
No formal education	16 (53.3)
Primary	9 (30.0)
Post primary	5 (16.7)
Living in school or not	
Yes	29 (96.7)
No	1 (3.3)
No of years in school	
<3 years	15 (50)
3–5 years	15 (50)

### Measures

#### Matson evaluation of social skills for individuals with severe retardation (MESSIER)

The MESSIER [40] is an 85-item semi-structured instrument designed to assess social skills and social behaviour in individuals with intellectual disability. The scale consists of six clinically derived dimensions; positive verbal (e.g., apologizes for unintentional mistakes), positive nonverbal (e.g., smiles in response to positive statements), general positive (e.g., responds appropriately when introduced to strangers), negative verbal (e.g., speech shows no emotion), negative nonverbal (e.g., responds to hugs with rigidity), general negative (e.g., has trouble waiting for needs to be met).

Informants are instructed to rate each item’s occurrence on a Likert-type scale, with ‘never’, ‘rarely’, ‘sometimes’, and ‘often’ The negative subscale score was calculated by reverse coding all items on the negative verbal, negative nonverbal, and negative general subscales. Also the items of the positive verbal, positive nonverbal, and positive general subscales were added together to generate the positive subscale. Total score of the MESSIER was calculated by adding the scores on the positive and negative scales together. The MESSIER has been shown to have good psychometric properties, with an internal consistency of r = 0.94, inter-rater of r = 0.73 and test–retest reliability of r = 0.86 after 2–3 weeks [7]. Cut-off scores of >151, 111–151 and <111 indicate no/minimal impairment, moderate impairment, and severe social skill impairment respectively [40, 41].

#### *Explore* social skills curriculum

*Explore* social skills curriculum [42] is an intervention programme for young people with developmental disabilities. It focuses on 50 important skills in ten domains such as ‘peer relationship’, ‘on the way to school’, important skills and vocational skills. The curriculum was developed in 2012 for use in schools for children and adolescents with special needs in the USA. The children are in inclusive education setting but have the *Explore* social skills curriculum utilized for their instruction. It provides pupils with step-by-step instruction through video modelling, photo-based directions, and role playing. The curriculum was adapted to the social and cultural context before its use in the study.

#### Adaptation process of the *Explore* curriculum for use by teachers

Permission to adapt the *Explore* curriculum was obtained from the Publisher [Attainment Company Incorporated, http://www.attainmentcompany.com].

A series of step-wise activities were conducted to achieve the goal of adaptation of the *Explore* curriculum into the social and cultural context. Firstly, an introduction and a preliminary review of the *Explore* curriculum to identify areas requiring specific attention was conducted. Sections from the curriculum were discussed as a group consisting of two researchers and six teachers; this served as a template for the teachers who suggested changes. Secondly, a meeting to reconcile the different suggestions was held and the teachers and the researchers agreed upon the different changes. These changes were made based on relevance of items to study setting and cultural considerations. Finally, a day training on the use of the adapted curriculum was held with the teachers; the training included practical demonstrations of lessons from the curriculum followed by demonstrations by the teachers. All teachers participating in the intervention were provided with free copies of the adapted *Explore* curriculum. Teachers were also provided with an overview of the curriculum and the aim of the study. They participated in practical sessions of some selected lessons from the curriculum.

A total of 15 lessons from the ten domains of the adapted curriculum were then selected, to be covered within the 8 weeks of the study, at an average of two lessons per week. The teachers and investigators taking into consideration priority social issues within the context, selected the 15 lessons. As a fidelity measure, the teachers’ coverage of the 15 lessons of the *Explore* curriculum was estimated by the recording of the duration of lessons and the extent to which each of the 15 lessons was taught. Based on these criteria, coverage of lessons among teachers was estimated to range from 60 to 85 %.

#### Study procedure

The study took place between October 2013 and March 2014. The intelligent quotient (IQ) of the participants was assessed using the Wechsler Intelligence Scale for Children, Fourth Edition (WISC-IV) [43] by the first author, who had been trained in its use. An average of 90 min was spent assessing each participant on the WISC-IV.

At baseline, socio-demographic information were obtained about each pupil directly from their hostel caregivers. The caregivers were also interviewed about the pupils by authors using the Matson Evaluation of Social Skills for Individuals with Severe Retardation, except for the only pupil in the day service whose demographic and MESSIER information was obtained from the class teacher. Information about participants’ parents was retrieved from the records obtained at the point of admission to the school.

Participants received lessons in their classrooms from the adapted *Explore* curriculum three times a week; with each lesson lasting for 45 min. Each lesson consisted of an introduction of the topic of discussion, self-talk story, where the teacher gave a narrative overview of the topic, and role-plays. Activities within each lesson allowed for the pupils to play active roles through effective systems of communication and social interaction. The teachers and investigators facilitated the role-plays. For example, a lesson on meeting a new person would place a pupil in either a new or old student role as they worked through meeting a new person. Participants received lessons from the curriculum for 8 weeks (February–March), at the end of March; post-intervention data was gathered using the same procedures that were used at baseline.

#### Data analyses

The data obtained was entered into the Statistical package for Social Sciences (SPSS) version 20. The paired t test was used to determine the difference between the pre-intervention and post-intervention scores on the social skills of the participants. The significance of the changes within categories of social skills impairment between pre and post intervention was tested using the Wilcoxon signed-rank test. Pre and post intervention analysis of MESSIER scores across socio-demographic variables was investigated using the Mann–Whitney U Test and the Kruskal–Wallis Test. Pearson correlation coefficients were used to evaluate the associations between IQ scores and social skills pre as well as post intervention. A fidelity analysis was done using multivariate analysis of variance (MANOVA) to analyse the gain scores of these groups on MESSIER using class as the independent variable. Reliable change index (RCI) was calculated using the methodology outlined by Jacobson and Truax [44]; the difference between the pre and post intervention data of the participants was calculated and divided by the standard error of the difference. The result obtain from this calculation is considered reliable if it is equal to or greater than 1.96. The standard error, and thus the index, depends on the standard deviation of the pre–post difference and on the reliability of the measure. Level of significance was set at p < 0.05.

#### Ethics approval and consent to participate

Ethical approval to conduct this study was obtained from the Oyo state Ethical Review Committee and permission to carry out the study was also obtained from the Oyo state Ministry of Education. Written consent containing information about the study was obtained from the participants’ parents or caregivers before the study. The study was also explained to the participants.

## Results

### Demographic and IQ characteristics of the participants

Table 1 shows the socio-demographic characteristics of the participants. Their ages ranged from 12 to 19 years with a mean age of 15.70 ± 1.89 years. There were 16 males (53.3 %) and 14 females (46.7 %). Ten of the participants (33.3 %) were from polygamous family settings (father had more than one wife) and the remaining 66.7 % were from monogamous settings. Sixteen (53.3 %) of the participants had mothers with no formal education while 9 (30 %) and 5 (16.7 %) had mothers with primary and post primary education respectively. About a third (36.7 %) of participants’ parents were not married. Almost all the participants (96.7 %) lived in the boarding facility and the mean number of years they had spent in school was 2.46 (±1.47) years.

Table 2 shows the distribution of the participants’ scores on both the full and sub-scales of WISC-IV. Their full IQ scores ranged from 40 to 56. The highest mean score was on the Perceptual Reasoning Index sub-scale (56.73 ± 8.78) while the lowest mean score was on the Verbal comprehension index sub-scale (50.30 ± 5.11).

**Table 2:** Distribution of IQ scores on the full and sub-scales of the WISC-IV (N = 30)

WISC scales	Mean scaled score	Mean IQ score	Maximum score	Minimum score
Full IQ	19.5 ± 7.9	44.0 ± 4.6	56	40
Verbal comprehension index	5.2 ± 2.1	50.3 ± 5.1	61	45
Working memory index	3.0 ± 1.4	52.2 ± 3.3	65	50
Perceptual reasoning index	8.9 ± 4.4	56.7 ± 8.8	79	45
Processing speed index	3.2 ± 1.8	53.7 ± 5.2	70	50

### Pre and post-intervention levels of the social skills impairment of the participants on the MESSIER

**Fig. 1:**
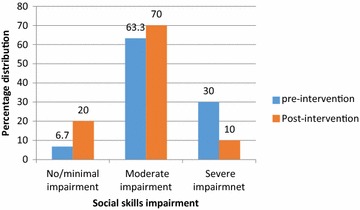
Pre and post-intervention levels of the social skills impairment of the participants on the MESSIER

Figure 1 shows the social skill categories of the participants on the MESSIER questionnaire. Eighteen of the participants (63.3 %) had moderate social skills impairment, 2 (6.7 %) had none or minimal impairment and 10 (30 %) had severe impairments at baseline. At the end of the intervention, there was a 20 % reduction in the number of participants in the severe social skills impairment category and 13.3 % increase in the number of participants in the ‘none or minimal’ social skills category. Wilcoxon signed-rank test shows that the change within the categories of social skills was statistically significant (Z = −2.887; p = 0.004).

Using standard deviation difference of 2.90 and a standard error of 4.39 (based on the pre and post intervention scores on the MESSIER) and the reported internal consistency reliability of 0.94, we calculated the reliable change index (RCI) of 12.16. Based on this RCI, 12 participants (40 %) showed reliable improvement, 2 (6.7) showed reliable deteriorated and the remaining 16 (53.3 %) showed no reliable change.

### Difference between pre and post-intervention scores on MESSIER scales

Table 3 shows the difference in the pre and post-intervention social skills total scores of the participants. The mean pre and post-intervention total scores were 126.63 ± 17.91 and 135.97 ± 20.81 respectively with a mean difference of 9.34 (t = 3.71; p = 0.001). There was also a statistically significant difference in the general positive sub-scale (t = 2.87; p = 0.008).

**Table 3:** Difference between pre and post- intervention scores on MESSIER scales (N = 30)

MESSIER scale	Pre-intervention mean (SD)	Post-intervention mean (SD)	Mean difference	t	p
Positive verbal	11.9 (5.3)	14.4 (6.6)	2.57	1.94	0.063
Positive nonverbal	22.7 (8.1)	21.8 (7.5)	0.97	0.98	0.338
General positive	33.8 (12.2)	37.4 (10.2)	3.53	2.87	*0.008*
Total positive	68.1 (23.9)	73.2 (19.0)	5.10	1.92	0.364
Negative verbal	17.4 (5.8)	18.8 (9.0)	8.71	0.92	0.364
Negative nonverbal	19.8 (7.6)	22.6 (10.1)	2.80	1.60	0.121
General negative	18.8 (6.0)	19.47 (7.2)	0.67	0.66	0.517
Total negative	57.0 (16.7)	59.9 (19.4)	2.90	0.90	0.378
Overall total	126.6 (17.9)	136.0 (20.9)	9.34	3.71	*0.001*

The significant value (p < 0.05) is in italic

*MESSIER* Matson evaluation of social skills for individuals with severe retardation

### Pre and post-intervention analysis of social skills by socio-demographic characteristics

Table 4 shows the pre and post-intervention analysis of social skills by socio-demographic characteristics using the Mann–Whitney U and Kruskal–Wallis tests. The results show that the distribution of the mean difference between pre and post intervention scores differs significantly (p = 0.015) across the categories of mother’s level of education, with participants whose mother had no formal education having lower mean score difference (12.86 ± 8.03) than those with either primary (18.90 ± 12.33) or post primary (16.30 ± 9.60) education. There was no statistically significant difference in the distribution of the mean differences across other socio-demographic variables including age and gender. In addition, Pearson correlation coefficients were used to evaluate the associations between IQ scores and social skills pre as well as post intervention. There was a moderate negative correlation between IQ scores and pre-intervention MESSIER scores [r(28) = −0.43, p = 0.007] which means that the higher the IQ scores the more severe the social skills impairment because lower MESSIER score connotes more impairment and a weak positive correlation between IQ scores and post-intervention MESSIER scores [r(28) = 0.37, p = 0.012].

**Table 4:** Mean difference between pre and post-intervention analysis of social skills by socio-demographic variables (N = 30)

Socio-demographic variable	Mean difference (between pre and post scores)	p
Sex		
Male	14.88 ± 9.29	0.790^a^
Female	16.71 ± 11.78	
Religion		
Christianity	16.48 ± 11.28	0.737^a^
Islam	13.29 ± 6.73	
Family type		
Monogamy	17.75 ± 11.72	0.650^a^
Polygamy	11.70 ± 5.46	
Marital status of parents		
Married	10.00 ± 15.72	0.471^a^
Not married	12.00 ± 16.70	
Father’s level of education		
No formal education	13.06 ± 6.03	0.805^b^
Primary	14.44 ± 10.99	
Post primary	26.60 ± 14.94	
Mother’s level of education		
No formal education	12.86 ± 8.03	*0.015* ^b^
Primary	18.90 ± 12.33	
Post primary	16.30 ± 9.60	

^a^Mann–Whitney U test

^b^Kruskal–Wallis test

The significant value (p < 0.05) is in italic

### Fidelity analysis

Fidelity analysis was carried out to evaluate the extent to which teachers’ coverage was associated with pupils’ gains by creating groups based on overall teacher reported coverage of lessons from the curriculum. There were three groups: Group 1 included students from class A where teacher reported 85 % coverage, Group 2 included students from class B where teachers reported 70 % coverage and 60 % coverage was reported by teachers in class C constituting Group 3. A multivariate analysis of variance (MANOVA) was used to analyse the gain scores of these groups on MESSIER using class as the independent variable. Descriptive analysis on the MANOVA showed that Group A had the highest mean gain (27.88). Although the overall MANOVA was not significant; Wilks Lambda = 0.313, F = (2, 27) = 0.787, p = 0.611, the effect size for Group A (partial η^2^ = 0.405) and Group B (partial η^2^ = 0.373) were within the medium range.

## Discussion

The study aimed at investigating the changes in the social skills of pupils with intellectual disability using an adapted version of the *Explore* social skills curriculum. In keeping with growing evidence that social skills can be improved through classroom-based interventions [44], the findings from this study suggest that the social skills of the pupils with intellectual disability improved with the intervention.

There are interesting aspects of the socio demographic characteristics of participants in this study. The parents of adolescents in this study had a divorce rate (36.7 %) over three times higher than the rate of 11 % obtained in a study of adolescents in mainstream schools in the same community as the current study [44]. It has been documented that chronic childhood illnesses and the presence of a child with disability are major sources of family distress and dysfunction leading to parental divorce [45], this might be the reason for this higher divorce rate recorded in our study. Also, majority (53.3 %) of the mothers of adolescents in this study had no formal education. In the region of Nigeria where this study was carried out, it has been documented that up to 61 % of women often do not receive any formal education [46]. Hence the mothers of children in this government owned facility for adolescents with intellectual disability had no formal education. The school is owned by the government and offers free education to attendees and the hostel accommodation is also highly subsidized by the government. It is therefore likely that the lower socioeconomic classes, who are also less likely to be educated, would access this facility. This services offered in the school are very basic and lacking in quality and hence, the better educated in society would more likely access private and better resourced facilities for their children with intellectual disability. A study of obstetric risk factors and subsequent mental health problems in a hospital in Southwest Nigeria, revealed that children with intellectual disability were more likely to have suffered birth injury [47]. In addition, children who suffered birth injury were more likely to have a parent in an unskilled occupation [47]. Studies in low-income settings reveal that lack of formal education of mothers’ correlates with poor utilization of ante-natal care, increased risk of complications like asphyxia and brain infection in the baby that can subsequently lead to intellectual disability [47, 48].

All participants in our study except one were living in the boarding house; this finding is a reflection of what happens to children with intellectual disability in the developing world. Cultural beliefs and attitudes about children with disability and their family are still largely negative. In many parts of Africa, children with disability are seen as “objects of shame”, who should be hidden away [49, 50]. This is coupled with the fact that there are no disability benefits or support from social welfare systems and hence parents, especially mothers of children with disability often report significant stress associated with caring for these children [51]. Formal settings like boarding schools, such as the school of current study or informal settings such as religious settings are often sought out for respite care or long stay institutionalizations [49].

All the participants scored below 69 on the test of intelligence and this score is defined as extremely low IQ on the WISC category. This is not surprising, according to the International Classification of Disease (ICD-10), an IQ below 70, in addition to assessment of adaptive skills, is an important criterion that is required for making the diagnosis of intellectual disability [52]. Moreover, studies among children and adolescents with intellectual disability attending special schools have consistently found low IQ scores as compared with those in inclusive educational settings [53]. The study site currently uses ‘educable’, ‘trainable’ and ‘profound’ mentally retarded to designate the different groups in the school. In the last two decades, the field of intellectual disability has experienced a lot of scrutiny in terms of appropriate terminologies and classifications, with most of the scrutiny coming from the high income countries [54]. Globally, the terminology ‘mental retardation’ is gradually being replaced with ‘’intellectual disability’ as a result of the current understanding of the concept of disability has a human phenomenon with its origin in organic and/or social factors as opposed to the earlier description of it as a deficit [55, 56]. The word retarded is often now used interchangeably with words like disdain or stupidity, and often does communicate derogation or disrespect [57]. Educationally, children with ID are classified into three categories; educable mentally retarded, trainable mentally retarded and severely or profoundly mentally retarded [58]. According to Krainz [59], the “educable” describes those children with IQ scores between 50 and 75, these children are believe could benefit from an education while the “trainable,” children usually scored between 30 and 49 on IQ tests. Any child scoring below 30 on the IQ examination was labelled as severely or profoundly mentally retarded and was considered “untrainable” and “totally dependent”. Although these designations appeared to provide ease of grouping to the teachers and help them to draw symbolic boundaries between individual students or groups [60], they however, have the tendency to limit the range of interventions and care each child receives. It has been documented that designations and terminologies can have significant consequences and limitations on the recipients of those designations [55]. For example it can affect their eligibility or ineligibility to be part of a service [55], or whether they are included or not included in a benefit such as protections against discrimination. These limitations could be more marked in settings where resources are scarce and where there are tendencies to concentrate the available resources on children with ‘lesser degree of disability’. It is therefore important that measures need to be put in place to ensure that some of the children especially those who are categorised as ‘profoundly mentally retarded’ are not neglected.

In the current study, virtually all participants had severe and profound levels of social skills impairment at baseline. The association between intellectual disability and social skills impairment is well established across the life cycle [61, 62]. A study that assessed the social skills of 100 adults with intellectual disability in two state-run facilities located in the South-eastern region of the United States, using the MESSIER found that participants had significant impairment in all the subscales of the MESSIER [3].

Although our study did not investigate the reasons for impaired social skills in the participants but some of the reasons alluded to the close relationship between impairments in social skills and intellectual disability are that individuals with intellectual disability often have problems detecting and understanding contextual clues and situations, identifying emotional and social relationships, and understanding others’ feelings and perceptions.

A key finding from our study was the significant difference in the mean change between the pre- and post-intervention scores on the social skills scales after 8 weeks of classroom-based intervention. In a randomised control trial of 222 adolescents with developmental disabilities in the United States the application of a social skills training programme called, “Working at Gaining Employment Skills (WAGES)” obtained significant improvements in the mean score difference between pre and post assessments of the adolescents on the Social Skills Rating System (SSRS) in the intervention group (Mean difference = 1.52) when compared to a control group (Mean difference = 0.51) [25]. A systematic review of ten studies on social skills interventions in adolescents and young people with disabilities found a significant (z = 4.61, p = 0.001) effect size (2.25) and concluded that social skills interventions are effective in this population [63].

Although our study find statistically significant difference in the social skills scores of the participants, however, it was difficult to measure the clinical evidence of this change; the absolute pre- and post-intervention scores were both within the moderate impairment in social skills. This is not surprising because the duration of the intervention was short and longer period of intervention is needed to record significant clinical evidence of this change in social skills. The traditional reliable change index provides one criterion by which the clinician may establish whether or not an observed change following an intervention is not due to chance [64]. The authors added a cautionary note that the RCI is never a sufficient index to demonstrate clinical improvement. They however explained that RCI provides information regarding the variability in treatment response from person to person. It also ensures that the degree of change was of sufficient magnitude to exceed the margin of measurement error [65]. These two latter points are limitations prevalent in statistical methods investigating changes resulting from intervention between groups of treated clients.

The cut-off criterion defined as leaving treatment in a normal state has been described as a more powerful determinant of Reliable and Clinical Significance Index (RCSI) than the reliable change index which is changing to a degree not attributable to chance [66]. Reaching a normal functioning in certain situations is not feasible, either because the disorder is incurable, for example in the case of intellectual disability or the current treatment technology is limited.

Interestingly, the correlational analysis between IQ sores and pre-intervention social skills scores showed that participants with higher IQ scores had more social skills deficits. Greater severity of ID has been associated with more impairment in the range of communication skills, hence, there is a significant reduction in both positive and negative behaviours [41]. Individuals with higher IQ score, are able to display a wider range of communicative and verbal behaviours, although a significant part of it is negative [41]. As a result, carers are more likely to report more difficulties in social interaction in those with higher IQ than those with lower IQ scores who tend to display reduced capacity for both verbal and nonverbal skills.

The finding of improvements in social skills with the *Explore* curriculum is important because studies have shown higher incidences of challenging behaviours and psychopathology in young people with intellectual disability [67, 68]. Studies reveal that about 30–60 % of persons with intellectual disability have a diagnosable mental disorder [69–71] and reasons for the higher incidence include poorer coping skills, ability to manage stress, problem-solving and conflict resolution skills [19]. One way of reducing challenging behaviours in these individuals is to improve their social skills.

Expectedly, the results of the fidelity analysis indicated that students with higher levels of teacher-rated coverage had greater gains in social skills. Another explanation for the greater gain aside the higher coverage might be because the participants in class A, where the highest gain was recorded, had the least disability. However, these two factors might be interrelated; higher teacher coverage could be due to the fact that participants in this class had lesser degree of disability in the first place. A more significant finding in this study is the fact that the participants in the other classes with more severe forms of disability also had appreciable changes in their social skills at the end of the 8-week intervention. This suggests that greater changes might have been recorded with a longer duration of the intervention; this finding is important for policy and planning in settings where there is a dearth of information on effective interventions for individuals with intellectual disability.

Fidelity of implementation is traditionally defined as the determination of how well an intervention is implemented in comparison with the original programme design during an efficacy and/or effectiveness study [72, 73]. Mowbray et al. [72] described two groups of criteria that are important in measuring fidelity of implementation. The first group is fidelity to structure; this includes adherence that describe whether the components of the intervention are being delivered as designed and the duration, which is the number, length, or frequency of sessions implemented. The second group of criteria is known as fidelity to process; this includes quality of delivery which is defined as the manner in which the implementer delivers the programme using the techniques, processes, or methods prescribed [72, 73]. Programme differentiation is whether critical features that distinguish the programme from the comparison condition are present or absent during implementation and participant responsiveness which is the extent to which participants are engaged by and involved in the activities and content of the programme. This study did not measure fidelity to process because the criteria for measuring fidelity to process are fundamentally difficult to quantify as they measure dynamic qualities of the intervention [74, 75] and measuring these criteria are resource-intensive [76]. In addition, in the unique population studied, assessing participants’ responsiveness, as fidelity measure would be a challenge due to their disabilities. There is often a legitimate need to tailor a programme model to local circumstances and resources and to the social and cultural needs of participants [77] and that is what was done in this study. With more elaborate support in terms of funding, future studies should be able to tackle many of these challenges and come up with a more detailed measure of effectiveness.

The findings in this study are important because they reveal that social skills of young people with intellectual disability can be improved over a relatively short period of time through teacher mediated classroom-based interventions. This is especially useful for a low-resource setting like the study site where the concept of task shifting is important to enable persons with developmental disabilities access services [78]. The participants also benefited from the mental health promotion that accompanies the social skills training. The teachers trained in the use of this curriculum and would continue to use the skills after the completion of the study to the benefit of the participants and others who would attend the school.

The findings from this study would be useful for advocating for and implementing inclusive education in a region where a large proportion of young persons with developmental disabilities are locked away in institutions, abandoned by their parents, or simply kept behind doors at home without any form of access to education or health services [79].

Sub-Saharan Africa is home to a large number of young people with intellectual disability [37]. A major challenge for young people with intellectual disability is a lack of access to education [37, 38]. Many young people with intellectual disability in sub-Saharan Africa are kept away in institutions and those who access education have to attend segregated schools [79]. Apart from stigma and discrimination against persons with intellectual disability, an important reason for the lack of inclusion education in sub-Saharan Africa inadequate training of teachers and political will. Findings from this study can be used to generate further research on the development of appropriate curricula for young persons with intellectual disability.

The findings are also relevant for policy making and designing educational packages for individuals with intellectual disability. Article 24 of the United Nations Convention of the Rights of Person with Disabilities, states that children with disabilities should be able to participate in the general education system and be educated in mainstream schools [80].

Currently, there are no structured educational plans and packages for this population in most parts of sub-Saharan Africa. For instance in Nigeria, teachers in schools for persons with intellectual disability are provided with the same curriculum as for mainstream schools. The mainstream school curriculum caters for core academic content areas like writing, reading and mathematics and fails to address issues such as adaptive functioning [81]. Therefore, it is important to investigate the possibility of replicating this study on a larger scale so the findings can be used in making a case for the development and integration of social skills training curricula for the use of young people with disabilities in schools. It is important to note that the training of teachers in the school was out-dated as well as the criteria and terminology used to place children in classes ‘educable’, ‘trainable’ and ‘profound’ mentally retarded. The evident need for the training of teachers in Southwest Nigeria is not limited to this school for children and adolescents with special needs. In a needs assessment for a school mental health programmes in rural and urban Southwest Nigeria, 56 primary school teachers were asked the question: ‘What comes to your mind when you hear about ‘mental illness’ or ‘mental health problems’ in children’? The teachers used outdated terms and words like ‘imbecile’, ‘insane’, and ‘moron’ in response to this question [82]. The need for training is urgent.

Although, the findings from this study contribute to existing knowledge, it is important to recognise that the study has several limitations. First, the sample for the study was relatively small this limits the generalisation of the findings. Second, because of the small size of the school, randomization, which could have provided for more robust findings, was not possible, hence future efforts with larger sample size and a randomised design might allow for broad generalisation of the findings. A third limitation of this study is the fact that the MESSIER was originally designed to measure social skills among adults with ID. But considering that resources are scarce especially for individuals with disabilities in the study setting, sometimes instruments are used outside the range of the original design. A way forward is to consider a thorough adaptation of this and other related instruments for use in this age group. Another important limitation is that we relied on caregivers who were aware of the participants’ conditions for the outcome rating. The caregivers’ ratings, especially the post-intervention data may have been affected by their awareness of and familiarity with participants’ conditions. This problem can be resolved in the future using a multi informant approach including data from teachers, caregivers, parents and through the use of direct assessment.

## Conclusion

This study supports findings from previous studies showing the association between impaired social skills and intellectual disability among adolescents. A very important outcome from the study is the improvement in social skills after 8 weeks of a teacher facilitated classroom-based intervention. The results from this study have the potential to influence educational policy thereby allowing for inclusive education of young people with intellectual disability in low-income settings.

## Abbreviations

ID: intellectual disability
IQ: intelligence quotient
MESSIER: Matson evaluation of social skills for individuals with severe retardation
WISC-IV: Wechsler intelligence scale for children-fourth edition
